# Magnitude of sedentary behavior and associated factors among secondary school adolescents in Debre Berhan town, Ethiopia

**DOI:** 10.1186/s12889-020-8187-x

**Published:** 2020-01-20

**Authors:** Osman Yimer Mohammed, Esubalew Tesfahun, Abdurahman Mohammed

**Affiliations:** 1 College of health Sciences, Department of Midwifery, Debre Berhan University, Debre Birhan, Ethiopia; 2 College of health Sciences, Department of Public Health, Debre Berhan University, Debre Birhan, Ethiopia; 3 College of health Sciences, Department of Nursing, Debre Berhan University, Debre Birhan, Ethiopia

**Keywords:** Sedentary behavior, Internet access, Social media, Maternal education

## Abstract

**Background:**

Sedentary life style is becoming increasingly common in this industrial age due to changes on the way people manufacture, transport and communicate. Sedentary lifestyle is associated with chronic diseases (diabetes, cardiovascular disease, and cancer), depression, obesity and premature mortality. The objective of this study was to assess the magnitude and associated factors of sedentary behavior.

**Methods:**

School based cross sectional study was conducted among 580 students from April 20 to May 10, 2019 in secondary schools in Debre Berhan City Administration. Sedentary behavior was measured using time spent on four activities (watching TV/Video, listening to music, surfing internet and playing games). Adolescents are considered sedentary if they spend two or more hours in one or all listed activities per day. Data was entered to Epidata version 4.2.2.1 and exported to SPSS version 20 for analysis.

**Result:**

A total of 580 (44.3% male and 55.7% female) students participated in this study. The magnitude of sedentary behavior (≥2 h per day) was 65.5% (95% CI = 61.32% - 69.08). Family monthly income greater than 8000 birr (AOR: 6.42, 95%CI = 2.18–18.78), maternal education (AOR: 5.12, 95%CI = 1.09–23.83), access to TV (AOR: 4.87, 95%CI = 1.99–11.87), access to mobile internet (AOR: 2.37, 95% CI = 1.14–4.93) and utilization of social media (AOR: 2.98, 95%CI = 1.43–6.17) were positively associated with adolescent sedentary behavior.

**Conclusion:**

The prevalence of sedentary behavior was high among adolescents of Debre Berhan town. Therefore, schools in the town should work towards creating awareness on the wise use of screen based entertainments.

## Introduction

Sedentary behavior is defined as any waking behavior characterized by an energy expenditure ≤1.5 METs while in a sitting or reclining posture [1]. Sedentary behavior includes television (TV) viewing, video game playing, computer use, reading, talking on the telephone, and sitting while commuting by automobile, bus, train, plane and ferry which all require an energy expenditure between 1.0 and 1.5 metabolic equivalent (METs) [2]. Sedentary behavior is very much influenced by industrial revolution which changes the way people manufacture, transport and communicate [2]. 23% of adults aged 18 years and above and 81% of adolescents (84% female and 78% male) are insufficiently physically active [3]. Screen time (ST) among high school students is higher compared with elementary [4]. Sedentary lifestyle is associated with ill health like chronic diseases (diabetes, cardiovascular disease, and cancer), higher depression and obesity [5–8]. Non-communicable diseases (NCDs) cause 70% of deaths globally, ranging from 37% in low-income countries to 88% in high-income countries [9].

Non-communicable diseases which includes heart diseases, various forms of cancer and diabetes mellitus claimed the life of 2.7 million people (28.6% of all deaths) in 2012 and 3.1 million people (33.5% of all deaths) in 2015 [10, 11]. In Ethiopia, non-communicable diseases such as cardiovascular diseases, diabetes mellitus and cancer caused 34% of all deaths in 2008 and 40% of all deaths in 2014 [12, 13].

Age, sex, socioeconomic status (higher family income), education (grade level), higher level of maternal education, parents’ occupation, media accessibility, experimentation with alcohol, being overweight are associated factors for sedentary behavior [4, 8, 14–20]. The magnitude of sedentary behavior and its correlates are well studied among developed and middle income countries. However, such studies are very rare in Sub-Saharan region. Similarly, the status of sedentary behavior and its associated factors are not well studied in Ethiopia. Furthermore, the gross school attendance ratio in Ethiopia is 91%. School attendance rate is high among adolescents in the urban area and the physical inactivity level is also high globally among these age group [3, 21]. Therefore, the objective of this study was to assess magnitude of sedentary behavior and its associated factors among adolescents of secondary schools in Debre Berhan City Administration, 2019.

## Methods and materials

### Study design and setting

A school based cross sectional study was conducted among secondary school adolescents in Debre Berhan City Administration from April 20 to May 10, 2019. Debre Berhan City Administration is the capital of North Shoa administrative zone of Amhara Regional state. It is located 695 km from Bahir Dar, the capital of Amhara Regional State, and 130kms from Addis Ababa, the capital of Ethiopia. The town has four governmental and one private secondary Schools. In 2018/19 academic year, there were 11,111 secondary school students in the town.

### Sampling procedures

Multistage stratified simple random sampling technique was used to select the study participants. Primarily, the schools were stratified into two strata, public and private schools. Then, two public and one private schools were first selected by simple random sampling. After that, the sample size was allocated to each high school proportional to the number of students in each schools. Similarly, the allocated number was also distributed to each grade level (grade 9, 10, 11 and 12) proportionally. Finally, students were selected using computer generated simple random sampling from each section.

### Operational definition

**Screen based sedentary time**: Time spend watching TV, Listening music, using Internet, playing mobile and computer game.

**Positive for Sedentary Behavior**: Adolescents who have ≥2 h average screen based sedentary time per day [2].

**Adolescent**: in this research are students between the age of 13 and 19.

### Data collection tool and procedures

A pretested, Interviewer Guided self-administered, structured questionnaire was used. Sedentary behavior was assessed using questions generated from the sedentary behavior questionnaire (SBQ), HELENA study sedentary questionnaire and different related researches [4, 22, 23]. Sedentary behavior was assed using four items which includes watching TV/Video, listening music, playing computer or mobile game and using internet. The internal validity of the items were assessed using Cronbach alpha test during the pretest (α-coefficients 0.76). Sociodemographic variables like sex, age, grade level, residence, housing type, parent education, household monthly income, parent occupation, access to TV, access to mobile-phone, access to internet, access to play ground and gymnasium were assessed. The English version of the structured questionnaire was translated into the local language, Amharic and back translated to English.

### Data processing and analysis

The collected data were checked manually at the site of data collection for completeness and consistency. The cleaned data were entered to Epidata version 4.4.2.1 software and exported to Statistical Package for Social Sciences (SPSS) version 20 software for analysis. Descriptive statistic was used to summarize the socio-demographic characteristics and sedentary behavior.

Sedentary status was determined by categorizing the average time spent on the four sedentary activities into < 2 h and ≥ 2 h [24, 25]. To identify associated factors to sedentary behavior, bivariate logistic regression was performed to each independent variable with the dependent variable. Those variables with *p* value < 0.2 in the bivariate logistic regression analysis were included in the multiple logistic regression. Strength of association was measured using odds ratio, and 95% confidence interval. Statistical significance was declared at p value < 0.05.

Screen based sedentary time was calculated by adding media related times (watching TV/Video, listening music, using internet and playing mobile and computer games); multiplying week day sum by five and weekend day by two and divide the sum of the two by seven. Adolescents with mean hours 2 and above were classified as sedentary.

## Results

### Socio-demographic characteristics

A total of 580 adolescents were participated in this study, which yields a response rate of 98.14%. The reason for non-participants were absenteeism at the time of data collection and reluctance to respond during repeated attempts made to communicate after the data collection time. Among the study participants, 257 (44.3%) were male and 323 (55.7%) were female with median age of 18 years (IQR: 16–18). About 215 (37.1%) were from grade 10 and 549 (94.4%) were Orthodox Christian (Table 1).

**Table 1:** Sociodemographic, Family Characteristics and Access to Media and Environmental Facility of Adolescents among high school students in Debre Berhan Town, Ethiopia, 2019

Variable	Category	Number	Percent (%)
Sex	Male	257	44.3
	Female	323	55.7
Age	14–14	160	27.6
	17–19	420	72.4
Grade	9	126	21.7
	10	215	37.1
	11	71	12.2
	12	168	29
Religion	Orthodox	549	94.7
	Muslim	12	2.1
	Protestant	11	1.9
	Other	8	1.4
Mother Education	No Education	184	31.7
	Elementary	188	31.2
	Secondary	88	15.2
	University/college	127	21.9
Father Education	No Education	144	24.8
	Elementary	180	31
	Secondary	91	15.7
	University college	165	28.4
Mother Occupation	House wife	341	75.9
	Private	129	17.2
	Government	101	6.9
	NGO	9	0
Father Occupation	Farmer	216	37.2
	Private	169	29.1
	Government	164	28.3
	NGO	31	5.3
Family Monthly Income	≥8000	260	31.4
	5001–7999	66	12.9
	2001–5000	126	24.7
	≤2000	158	31
Family Residence	Urban	362	62.4
	Rural	218	37.6
Residence House	Own	407	70.2
	Rent	154	26.6
	Condominium	19	3.3
Television	Yes	417	71.9
	No	163	28.1
Satellite Dish	Yes	345	59.5
	No	235	40.5
Computer	Yes	145	25.0
	No	435	75.0
Private Internet	Yes	18	3.1
	No	562	96.9
Own Phone	Ye	432	74.5
	No	148	25.5
Phone Internet (*n* = 432)	Yes	293	67.8
	No	139	32.2
Social media use	Yes	253	43.6
	No	327	56.4
Play Ground access	Yes	339	58.4
	No	241	41.6
Gymnasium access	Yes	106	27.6
	No	474	72.4

### Adolescent’s family socio-demographic characteristics

From the total families of the study participants 362 (62.4%) were from Urban provinces and 407 (70.2%) live in their own house, 184 (31.7%) mothers and 144 (24.8%) father of adolescents were not attended formal education. About 341 (75.9%) mothers were house wives and 216 (37.2%) fathers were farmers. Concerning family income, the median income was 5000 with the inter-quartile range (IQR) 2000–8000 (Table 1).

### Adolescents media access and characteristics of the living environmental

Among all adolescents who participated in this study 417 (71.9%) had access for television, 435 (59.5%) had access to satellite dish, 339 (58.4%) had playground around their residence and 106 (27.6%) had access for gymnasium. About 432 (74.5%) adolescents had access to mobile phone and 67.82% (293/ 432) of them had access for mobile internet connection. A total 253 (43.6%) used social media (Table 1).

### Sedentary time and behaviors

Among the total participants 387(65.2%) [28.38% male and 36.9% female] were found to be sedentary (95% CI = 61.32% - 69.08). The average screen based sedentary time was 3.3 h (3.3 + 2.29 SD). Watching TV/Video (1.22) contributed more time for sedentary behavior. All sedentary times spend doing different activities showed a slight increment at the weekend compared with the week days (Table 2).

**Table 2:** Time Spent doing different activities among Adolescents of high school students in Debre Berhan Town, Ethiopia, 2019

Sedentary Behavior	Mean week day (SD)	Mean week end day (SD)
Watching TV/Video	1.22(1.28)	1.5(1.6)
Listen Music	0.89(0.87)	1.01(0.97)
Play computer or mobile Game	0.48(0.64)	0.59(0.82)
Internet Use	0.52(0.79)	0.61(0.99)
Screen based sedentary time	3.12(2); (IQR = 1.25–4.25)	3.76(2.89) (IQR = 1.5–5.5)
Total screen based sedentary time	3.3(2.29), (IQR = 1.33–4.67)	

### Factors associated with sedentary behavior of adolescents among high school students in Debre Berhan town

Binary logistic regression was conducted between status of sedentary behavior and all dependent variables. Dependent variables with *P*-Value less than 0.2 like, grade, Family monthly income, mothers’ education, fathers’ education, mother’ occupation, father’ occupation, family residence, house status, access to TV, access to satellite dish, access to mobile internet and social media utilization were included to multiple regression analysis.

The result of multiple logistic regression model revealed that monthly income, maternal education, access to television, mobile internet and social media utilization found to be statistically significant. Accordingly, adolescents whose family earn more than 8000 per month were 6.42 (AOR 6.42, 95%CI = 2.18–18.78) times more likely to be sedentary compared with those whose families earn less than 2000 birr. Adolescents who have educated mother were 5.12 (AOR 5.12, 95%CI = 1.09–23.83) times more likely to be sedentary compared with those who have uneducated mother. Adolescents who have access to TV were 4.87 (AOR 4.87, 95%CI = 1.99–11.87) time more likely to be sedentary than those who do not have access to TV. Adolescents who used social media were also 2.98 (AOR 2.98, 95%C I = 1.43–6.17) times more likely to be sedentary than those who did not use social media (Table 3).

**Table 3:** Factors associated with Sedentary Behavior of adolescents among High school students in Debre Birhan, Ethiopia, 2019

Variables		Sedentary	Not Sedentary	COR	AOR(95%-CI)
Grade level	G = 9	73	53	1	1
	G = 10	124	91	1.95	0.39 (0.13–1.23)
	G = 11	59	12	0.54	1.22 (0.58–2.53)
	G = 12	122	46	1.93	0.82 (0.35–1.90)
Family Monthly Income	< 2000	66	92	1	1
	2001–5000	72	54	5.92	1.81 (0.64–5.17)
	5001–7999	44	22	3.94	1.45(0.52–4.04)
	> 8000	142	18	10.99	6.42 (2.18–18.78)***
Maternal Education	No Education	79	105	1	1
	Elementary	114	67	0.96	2.16(0.51–9.17)
	Secondary	76	12	3.56	5.12 (1.09–23.83)*
	College University	109	18	8.05	4.40 (0.842–22.99)
Father Education	No Education	51	93	1	1
	Elementary	108	72	1.12	0.85(0.23–3.10)
	Secondary	77	14	4.12	0.61 (0.14–2.69)
	College University	142	23	11.25	0.95 (0.19–4.85)
Mother Occupation	NGO	7	2	3.243	0.88 (0.10–7.39)
	Government	84	17	0.605	0.81 (0.09–7.04)
	Private	110	19	0.708	0.39 (0.04–3.64)
	House wife	177	164	1	1
Father Occupation	NGO	26	5	9.02	4.58 (0.58–35.92)
	Government	135	29	1.17	0.88 (0.13–5.80)
	Private	138	31	1.12	1.78 (0.27–11.75)
	Farmer	79	137	1	1
Family Residence	Urban	295	67	7.16	0.37 0.13–1.06)
	Rural	83	135	1	1
Housing condition	Own	293	114	2.71	0.75 (0.37–1.53)
	Rent	75	79	2.31	1.02 (0.18–5.69)
	Condominium	10	9	1	1
TV	Yes	330	87	9.09	4.87 (1.99–11.87)***
	No	48	115	1	1
Dish	Yes	289	59	7.54	1.132 (0.456–2.82)
	No	92	143	1	1
Computer	Yes	127	18	5.17	1.26 (0.46–3.44)
	No	251	184	1	1
Private Internet	Yes	16	2	4.42	0.62(0.09–4.07)
	no	362	200	1	1
Phone	Yes	301	131	2.12	1.33 (0.85–2.33)
	No	77	71	1	
Mobile Internet	Yes	236	57	2.37	2.37(1.14–4.93)*
	No	65	74	1	1
Social media	Yes	213	40	5.23	2.98 (1.43–6.17)**
	No	165	162	1	1

*Statistically significant *P* value < 0.05, ** *p* value < 0.01, *** *P* value < 0.001

NB: Model fitness and multicollinearity is checked

## Discussion

This study tried to measure the magnitude of sedentary behavior and associated factors. The prevalence of sedentary behavior was high (65.2%). A Number of factors were identified to be positively associated with sedentary behavior and they are consistent with past researches. These includes family monthly income, maternal education, access to media, access to mobile internet and social media utilization.

The prevalence of screen based sedentary behavior (≥2 h) was 65.5%. This was similar with study conducted in Brazil and Scotland which reported that the prevalence was around 69.2 and 68.6% respectively [14, 26]. The similarity may be due to the entertaining nature of media affects adolescents in the same way. This study finding was not consistent with other study in Canada, which reported the prevalence of sedentary behaviors to be 25.4% [27]. The inconsistency might be due to the difference on the measurement of sedentary behavior and the cut of point considered.

High socioeconomic status and high maternal education were associated with high screen time (> 2 h). This was consistent with studies conducted in Brazil. Accordingly, higher maternal education and higher family income were positively associated with more screen time [14, 18]. Another study in Ghana also reported that affluence was the main contributing factor for higher sedentary behavior [8]. This may be due to educated mother seeks information making them close to media so as their children and wealth makes media facilities accessible to the family [21, 28].

Access to media like TV was associated with high sedentary time. It was consistent with study in china which reported that media access was positively correlated with high screen time [4]. The similarity might reflect the similar effect of exposure to TV. Access to internet and use of social media were another significant predictor of sedentary behavior in our study. Age, grade level, occupation, residence, house status and access to satellite dish were not significantly associated factors.

## Limitation

The time measurement is merely dependent on the memory of participants and it might be wrongly estimated. The generalizability of this study for the country is limited since it was done in one region. Furthermore, we unable to compare with researches in the local and regional context due to lack of similar studies and difference in the operational definitions (definition of sedentary behaviors).

## Conclusion

The prevalence of sedentary behavior was high. Sedentary behavior (spending two or more hours with media related activity) was significantly affected by family monthly income, maternal education, access to TV, access to mobile internet and utilization of social media.

## Recommendation

To North Shoa and Debre Berhan City education office

- Better to avail facilities and mainstream the education on life style with the main academic program to enhance behavioral change

To secondary schools

- Better to create awareness regarding the recommended time to spend on screen based entertainment.

## Data Availability

The data set used in this study is available from corresponding author on reasonable request.

## Abbreviations

AOR: Adjusted Odds Ratio
CI: Confidence interval
COR: Crude Odds Ratio
IQR: Interquartile range
METs: Metabolic Equivalent
NCD: None communicable disease
SBQ: Sedentary behavior Questionnaire
SD: Standard Deviation
TV: Television
